# Consecutive multiscale feature learning-based image classification model

**DOI:** 10.1038/s41598-023-30480-8

**Published:** 2023-03-03

**Authors:** Bekhzod Olimov, Barathi Subramanian, Rakhmonov Akhrorjon Akhmadjon Ugli, Jea-Soo Kim, Jeonghong Kim

**Affiliations:** 1 AI Department, IT Convergence R &D Center, Vitasoft, Seoul, South Korea; 2 School of Computer Science and Engineering, Kyungpook National University, Daegu, 41586 South Korea

**Keywords:** Computational biology and bioinformatics, Engineering

## Abstract

Extracting useful features at multiple scales is a crucial task in computer vision. The emergence of deep-learning techniques and the advancements in convolutional neural networks (CNNs) have facilitated effective multiscale feature extraction that results in stable performance improvements in numerous real-life applications. However, currently available state-of-the-art methods primarily rely on a parallel multiscale feature extraction approach, and despite exhibiting competitive accuracy, the models lead to poor results in efficient computation and low generalization on small-scale images. Moreover, efficient and lightweight networks cannot appropriately learn useful features, and this causes underfitting when training with small-scale images or datasets with a limited number of samples. To address these problems, we propose a novel image classification system based on elaborate data preprocessing steps and a carefully designed CNN model architecture. Specifically, we present a consecutive multiscale feature-learning network (CMSFL-Net) that employs a consecutive feature-learning approach based on the usage of various feature maps with different receptive fields to achieve faster training/inference and higher accuracy. In the conducted experiments using six real-life image classification datasets, including small-scale, large-scale, and limited data, the CMSFL-Net exhibits an accuracy comparable with those of existing state-of-the-art efficient networks. Moreover, the proposed system outperforms them in terms of efficiency and speed and achieves the best results in accuracy-efficiency trade-off.

## Introduction

Recently, the amount of available data has considerably increased owing to the developments of Internet of Things, technological devices, and computational machines. Because of the widespread usage of these ubiquitous technologies, high volumes of various data, such as digital images, texts, speech, or various combinations of these, have been generated. Among the aforementioned types of data, images constitute a large portion of available data^1^.

Because of the accessibility of digital image data from cameras and sensors, these data need to be processed for analysis to obtain meaningful results. As digital image data are significantly large in volume and usually complex, sophisticated digital image analysis techniques, such as machine learning (ML) and deep learning (DL), have been used to efficiently handle them^2^. Several tasks have been performed to deal with digital images, such as image classification, semantic segmentation^3–5^, object detection^6,7^, and instance segmentation^8,9^. Image classification is crucial part of digital image analysis and a basic component of the other computer vision tasks because image classification models are used as a backbone for the abovementioned more advanced computer vision tasks^10,11^.

Image classification involves the extraction of useful features from a digital image and the classification of the image into one of the pre-defined classes based on the extracted features^12,13^. Manual verification and classification of digital images can be a laborious and monotonous process; thus, automating the image analysis process by using image classification methods is more efficient and less time-consuming^14,15^. Recent advances in these methods have facilitated the usage of image classification in several real-world applications, such as medical imaging^16,17^, face recognition^18^, human activity recognition^19^, and traffic control systems^20,21^.

Numerous studies have been conducted on the usage and importance image classification. Before the emergence of DL, several traditional methods were used to effectively analyze images. For example, some statistical methods, such as maximum likelihood, minimum distance, parallelepiped, are the most common traditional techniques for image classification^22,23^. Moreover, a few ML methods, such as k-nearest neighbors, support vector machines, and random forest, are used^24,25^. However, traditional image classification methods became obsolete after the introduction of DL methods, which are faster, more efficient, and more accurate. DL methods used for image classification already surpass human-level accuracy when abundant labeled data are available for training^26,27^.

However, manually labeling millions of available images is a time-consuming and laborious task; thus, obtaining a large number of manually annotated data for image classification model training is challenging^19,28^. Consequently, DL-based classification networks have limitations in learning useful features from labeled datasets with a limited number of images. The insufficiency of training data is apparent in many fields, such as medicine and fault detection^29,30^. The complex structure and large number of trainable parameters of state-of-the-art classification models^31–34^ often result in overfitting^35^. Additionally, the existing state-of-the-art classification networks cannot appropriately extract useful features from small-scale images and often exhibit poor performance on these data. Because the existing methods primarily focus on large-scale images and a prolonged training process, they typically leads to poor generalizability and unsatisfactory outcomes on small-scale images^12^. Although several lightweight models focus on efficient computation by reducing the number of trainable parameters^36–39^, they still encounter the underfitting problem3. These models cannot appropriately learn useful image features, leading to poor classification performance. Moreover, the existing DL-based image classification methods are not flawless or fast^40^; therefore, faster and more efficient, precise, and generalizable image classification models are being developed^41,42^.

By studying the currently available methods for image classification, we identified that these models can be improved in terms of accuracy and speed. Thus, in this study, we propose a novel image classification system called CMSFL-Net; it uses elaborate preprocessing and a carefully designed model architecture. The proposed model benefits from multiscale feature extraction and consecutive feature learning by using various feature maps with different receptive fields (RFs) to achieve better performance in terms of speed and accuracy when compared with the existing state-of-the-art methods. In general, the contributions of this study are as follows:

- The proposed method employs consecutive propagation of extracted features from various RFs, thus obtaining better classification accuracy using an efficient computation-based small-sized model.
- The proposed method utilizes an elaborate pre-processing stage and improved consecutive multiscale feature learning that enables it to achieve a better and faster training process.
- The proposed method exhibits high inference speed owing to an efficient computation-based lightweight model that uses few trainable parameters that allows the model to be used in real-time applications.
- The proposed method exhibits excellent generalizability and performance in limited, small-scale, and large-scale image datasets.
- The proposed method can be employed as a backbone model for other computer vision tasks, such as semantic segmentation, object detection, and instance segmentation, owing to its superiority in feature learning over the existing state-of-the-art DL-based classification models.

The remainder of the manuscript is organized as follows. Section 2 presents a thorough discussion on the existing methods for image classification and their weaknesses. Section 3 contains a thorough explanation of the proposed methodology. Section 4 provides detailed information on the conducted experiments and their results. Section 5 presents the experimental results. Finally, Section 6 concludes this study and defines future study topics.

## Related works

As discussed in Sect. 1, there has been a vast number of proposed traditional and DL-based approaches for image classification. In this section, we focus on only DL-based techniques since traditional approaches are not utilized with these data because of their poor speed and accuracy. The currently available DL-based approaches can be classified into computationally expensive-powerful and efficient-lightweight models.

### Computationally expensive and powerful DL-based models

One of the earliest and most powerful DL-based convolutional neural network (CNN) models for image classification is residual networks (ResNets)^31^. In this study, we proposed a model by reformulating the layers as learning residual functions with reference to the layer inputs instead of learning unreferenced functions. By employing the residual functions learning, the model is easy to optimize and obtains better accuracy from the increased depth of the network; consequently, it successfully addresses the problem of training deep neural networks (DNNs) and a vanishing gradient problem. Huan et al. proposed a dense convolutional network (DenseNet) that connects each layer to every other layer in a forward propagation^32^. The authors employed $L(L+1)/2$ direct connections instead of traditional L connections of networks with L layers. This direct connection allows the network to handle a vanishing gradient problem, ensures feature sharing, and significantly reduces the number of trainable parameters. Moreover, Xie et al. introduced a highly modularized DL-based classification model architecture that uses repetitive building blocks aggregating a set of transformations with the same topology and introduces “cardinality” dimension that serves as a crucial factor in addition to depth and width dimensions^33^. Alternatively, we further improved the ResNet using more convolutional operations with various filters while retaining the same computational complexity.

Moreover, Gao et al. proposed a novel building block for CNNs by constructing hierarchical residual connections within a single residual block^34^. The Res2Net represents multiscale features at a granular level and increases the RFs for each layer of the network. Mansilla et al. proposed a novel method that incorporates anatomical priors in the form of global constraints into the data learning process to boost the realism of the warped images after registration. The method learns global nonlinear representations of image anatomy using segmentation masks and uses them to constrain the registration step^17^. Oregi et al. developed a system to address an issue of adversarial attacks by extracting color gradient features from input images at various sensitivity levels to detect various manipulations. This technique employs a DCNN to classify an image, whereas a discrimination model analyzes the extracted color gradient features with sequence data to identify the legitimacy of input images^2^. Wei et al. formulated an interactive visual model that uses self-interaction, mutual interaction, multi-interaction, and adaptive interaction, forming the first interactive completeness of the visual interaction network. We also employ the adaptive adjustment mechanism to enhance the performance of the DCNN model. Although the aforementioned models achieve state-of-the-art performance in terms of accuracy in an image classification task, they suffer from inefficient computation, slow training, and inference speed due to an extensive number of trainable parameters and floating point operations (FLOPs). Also, the models experience poor generalizability for small-scale images since they cannot completely learn useful features from the images within a short period of training. Although DenseNet reduced the number of trainable parameters and modified versions of ResNets improved the feature extraction and accuracy, they are still significantly slower in comparison to the lightweight models that are introduced in the next subsection. Regarding Res2Net, it extracts the features from particles of the input features to every other layer rather than learning the features from initial inputs. Considering that the inputs to the next layers in the network lose information as the training continues, the model can only partially use the power of useful features from the original input, which leads to poor classification performance of the network.

### Efficient and lightweight DL-based models

ShuffleNet, MobileNet, and MnasNet are the most widely employed lightweight DL-based classification models. They are mainly used in devices with limited computational power due to their efficient computation and small memory requirement. ShuffleNet employs pointwise group convolution and channel shuffle that allows the model significantly reduces computational expenses while retaining a competitive accuracy^43^. Ma et al. further improved the original ShuffleNet by introducing ShuffleNet V2^37^. The model considers the indirect metric of computation complexity, such as FLOPs, and the direct metric, such as required memory and device characteristics.

Regarding the other efficient and lightweight model, MobileNet V1 employs a streamlined architecture, which utilizes depth-wise separable convolution operations to formulate a lightweight network architecture. The authors of the MobileNet V1 introduced two hyper-parameters that allow an engineer to select an appropriate model size based on the problem characteristics. The MobileNet V1 is still outperformed by standard CNN architecture-based models. Therefore, to address the issue, MobileNet V2 is proposed^44^. The model benefits from an inverted residual structure where the shortcut connections are between the thin bottleneck layer while the intermediate expansion layer employs depthwise convolution operation to filter features as a nonlinearity source. MnasNet is based on MobileNet V2 model architecture and introduces lightweight attention modules using squeeze and excitation into the bottleneck structure^38^. These structures are placed after the depthwise filters feed-forward pass to obtain attention to be applied to the largest image representation. Qian et al. improved the MobileNet V2 and proposed MobileNet V3 that uses modified swish nonlinearities by replacing the original sigmoid function with the hard sigmoid to alleviate the vanishing gradient problem and ensure better accuracy^39^. In general, lightweight models obtain a good trade-off between speed and accuracy; however, they exhibit poor feature learning ability than vanilla deep CNN networks. Consequently, these models cannot obtain desirable accuracy when trained using limited or small-scale image data, causing an underfitting problem.

To address the aforementioned problems of the existing powerful (parallel approach of feature extraction) and efficient models (underfitting for limited data), we propose a novel model that uses consecutive multiscale feature learning from the original input features and sequentially propagates these features to decrease the number of trainable parameters and model size. Moreover, the proposed method exhibits a simplified model structure that allows improved feature extraction that leads to better classification performance due to the usage of the consecutive feature learning method.

## Proposed methodology

**Figure 1:**
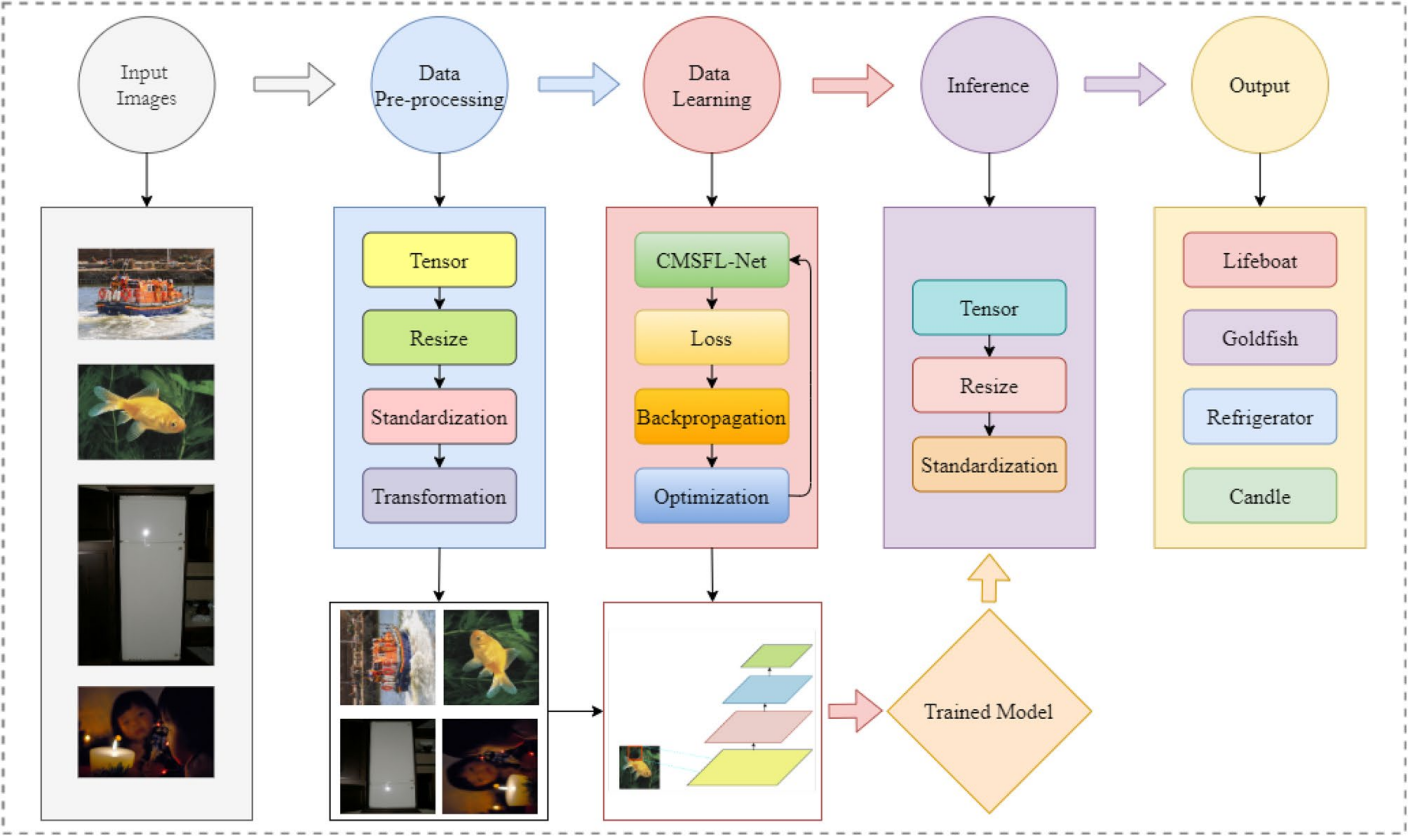
General overview of the proposed CMSFL-Net system.

In this section, we describe the proposed CMSFL-Net system in detail. An overall graphical illustration of the proposed method is illustrated in Fig. 1. Specifically, the CMSFL-Net contains three significant steps, which are data pre-processing, data learning, and inference.

### Data pre-processing

In the pre-processing stage, dataset images are represented as tensors to make the computation in the training process more convenient and efficient. Specifically, the images are extracted from directories and represented as tensors since they ensure more natural representations of multidimensional data. The resulting tensor is 4D—$X\in {\mathbb {R}}^{M\times {C}\times {H}\times {W}}$, where *M*, *C*, *H*, and *W* are the total number of images, number of channels, image height, and image width, respectively. After obtaining the images in tensors, they are resized to match the input size of deep CNN (DCNN) later in a data learning phase. Then, the resized image pixel values are standardized to follow a standard normal distribution using (1) as follows.

1 $$\begin{aligned} X_{std} = \frac{X - \displaystyle \frac{1}{M}\displaystyle \sum _{i=1}^{M} x_{i}}{\sqrt{\displaystyle \frac{1}{M}\displaystyle \sum _{i=1}^{M} {\left( x_{i} - \displaystyle \frac{1}{M}\displaystyle \sum _{i=1}^{M} x_{i} \right) }^2 }} \end{aligned}$$

In (1), *X* and $X_{std}$ are the original and standardized data; while *i* and *M* are a particular data point and the total number of instances, respectively. Notably, data standardization of validation and test data is performed using the training data distribution to avoid overfitting to the training set and increase the generalization ability of the DCNN model.

Finally, data augmentation techniques are applied to increase the number of images for better learning multiscale features and better generalization ability of the proposed model during training and inference, respectively. Based on the dataset images’ characteristics, we apply various image augmentation methods as follows:

2 $$\begin{aligned}{} & {} \left[ \begin{array}{ll} &{}x_{atr}\\ &{}y_{atr}\\ &{}1\\ \end{array}\right] = \left[ \begin{array}{lll} cos\alpha &{} -sin \alpha &{} 0\\ sin\alpha &{} cos \alpha &{} 0\\ 0&{}0&{}1\\ \end{array}\right] \left[ \begin{array}{ll} &{} x_{org}\\ &{} y_{org}\\ &{}1\\ \end{array}\right] \quad \quad \left[ \begin{array}{ll} &{}x_{atr}\\ &{}y_{atr}\\ &{}1\\ \end{array}\right] = \left[ \begin{array}{lll} s_{x} &{} 0 &{} 0\\ 0 &{} s_{y} &{} 0\\ 0&{}0&{}1\\ \end{array}\right] \left[ \begin{array}{ll} &{} x_{org}\\ &{} y_{org}\\ &{}1\\ \end{array}\right] \nonumber \\{} & {} \left[ \begin{array}{ll} &{}x_{atr}\\ &{}y_{atr}\\ &{}1\\ \end{array}\right] = \left[ \begin{array}{lll} -1 &{} 0 &{} 0\\ 0 &{} 1 &{} 0\\ 0&{}0&{}1\\ \end{array}\right] \left[ \begin{array}{ll} &{} x_{org}\\ &{} y_{org}\\ &{}1\\ \end{array}\right] \quad \quad \left[ \begin{array}{ll} &{}x_{atr}\\ &{}y_{atr}\\ &{}1\\ \end{array}\right] = \left[ \begin{array}{lll} 0 &{} 1 &{} 0\\ 1 &{} 0 &{} 0\\ 0&{}0&{}1\\ \end{array}\right] \left[ \begin{array}{ll} &{} x_{org}\\ &{} y_{org}\\ &{}1\\ \end{array}\right] \end{aligned}$$

Specifically, we employ affine transformations to rotate the 2D image dimensions along the *X* and *Y* coordinates, change the scale of the images using $s_x$ and $s_y$ parameters, and mirror the images across the *X* and *Y* axes.

### Data learning

After data preprocessing stages are completed, useful features of images are extracted using a consecutive multiscale feature learning-based model - CMSFL-Net.

#### Network architecture

**Figure 2:**
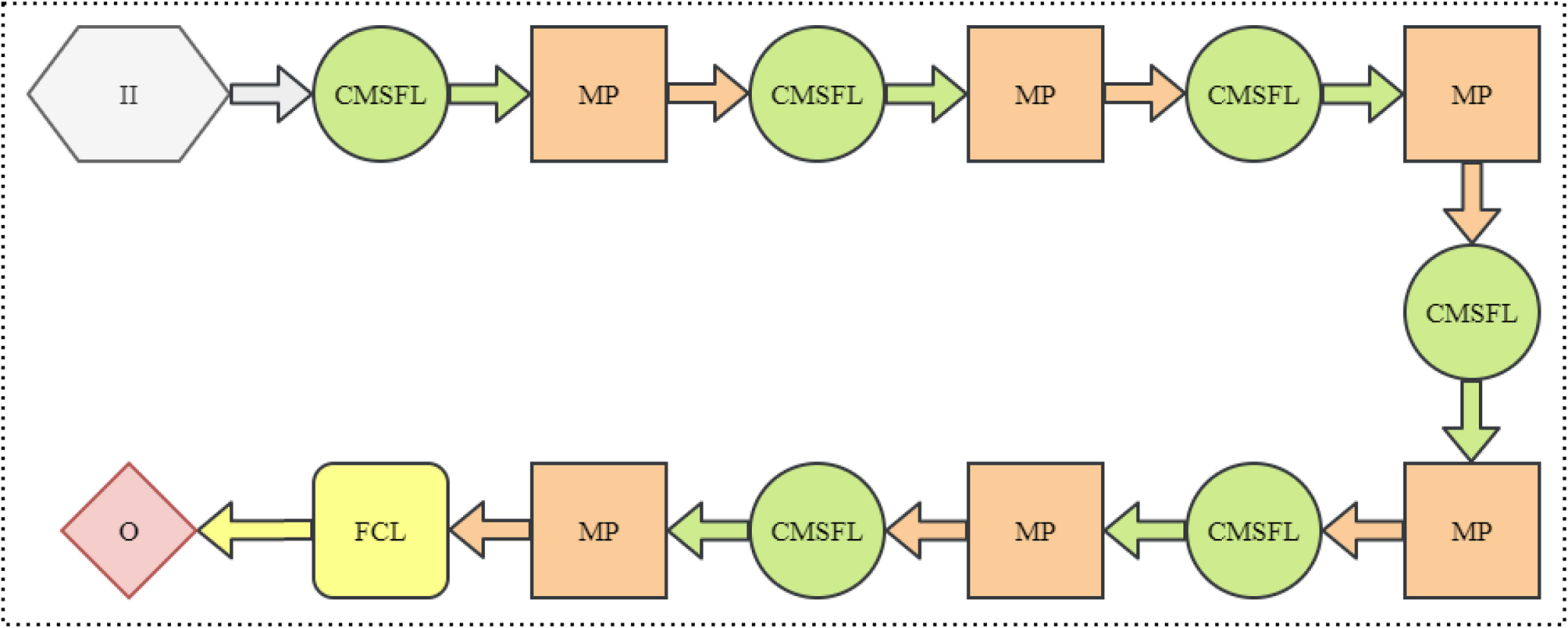
Detailed visualization of the proposed CMSFL-Net. II and O represent input image and output. MP and FCL stand for max-pooling operation, and a fully connected layer, respectively.

The model is a combination of consecutive multiscale feature learning (CMSFL) modules for extracting features from an image, a max-pooling operation for decreasing the spatial dimension of an image, and a fully connected dense layer for linearly classifying an image into one of the pre-defined classes based on the learned features in CMSFL modules inspired from^45–47^. The complete network architecture of the CMSFL-Net is provided in Fig. 2.

**Figure 3:**
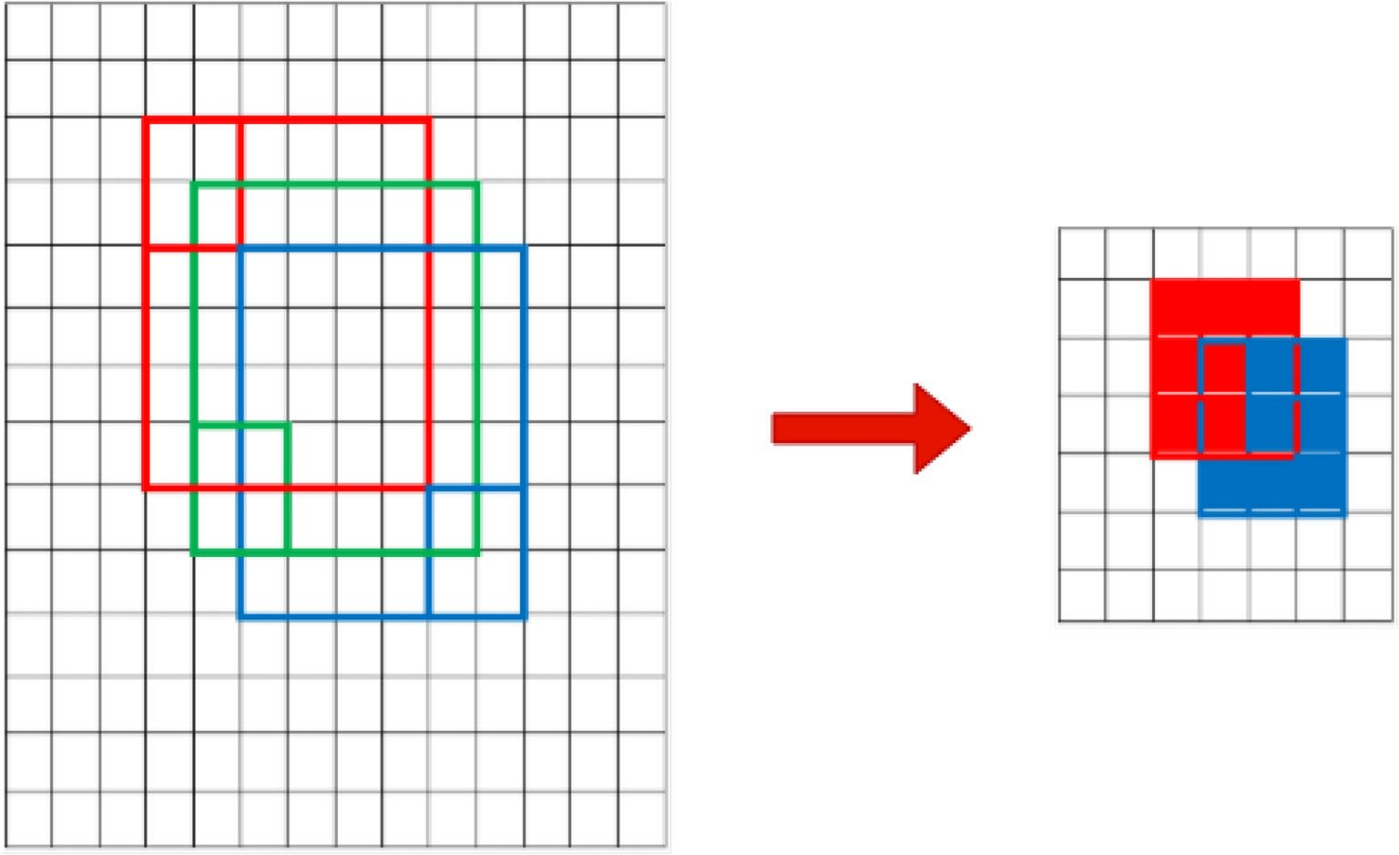
Graphical illustration of information loss problem resulted from applying a max-pooling operation in a DCNN model. Red, green, and blue boxes represent specific area of a region and 2 $\times$ 2 pooling kernel size. The output feature map on the left side contains no information from green-boxed area, which clearly demonstrates the information loss after applying the max-pooling operation.

As shown in Fig. 2, every CMSFL module is followed by a max-pooling operation, which decreases the spatial dimension of its input by a factor of two by retaining the most striking pixels with the highest value in comparison with the ones in its neighborhood. Despite being an efficient method to reduce the computational complexity of a DCNN model, max-pooling operation results in tremendous information loss^48,49^. The problem is illustrated in Fig. 3.

#### CMSFL module

**Figure 4:**
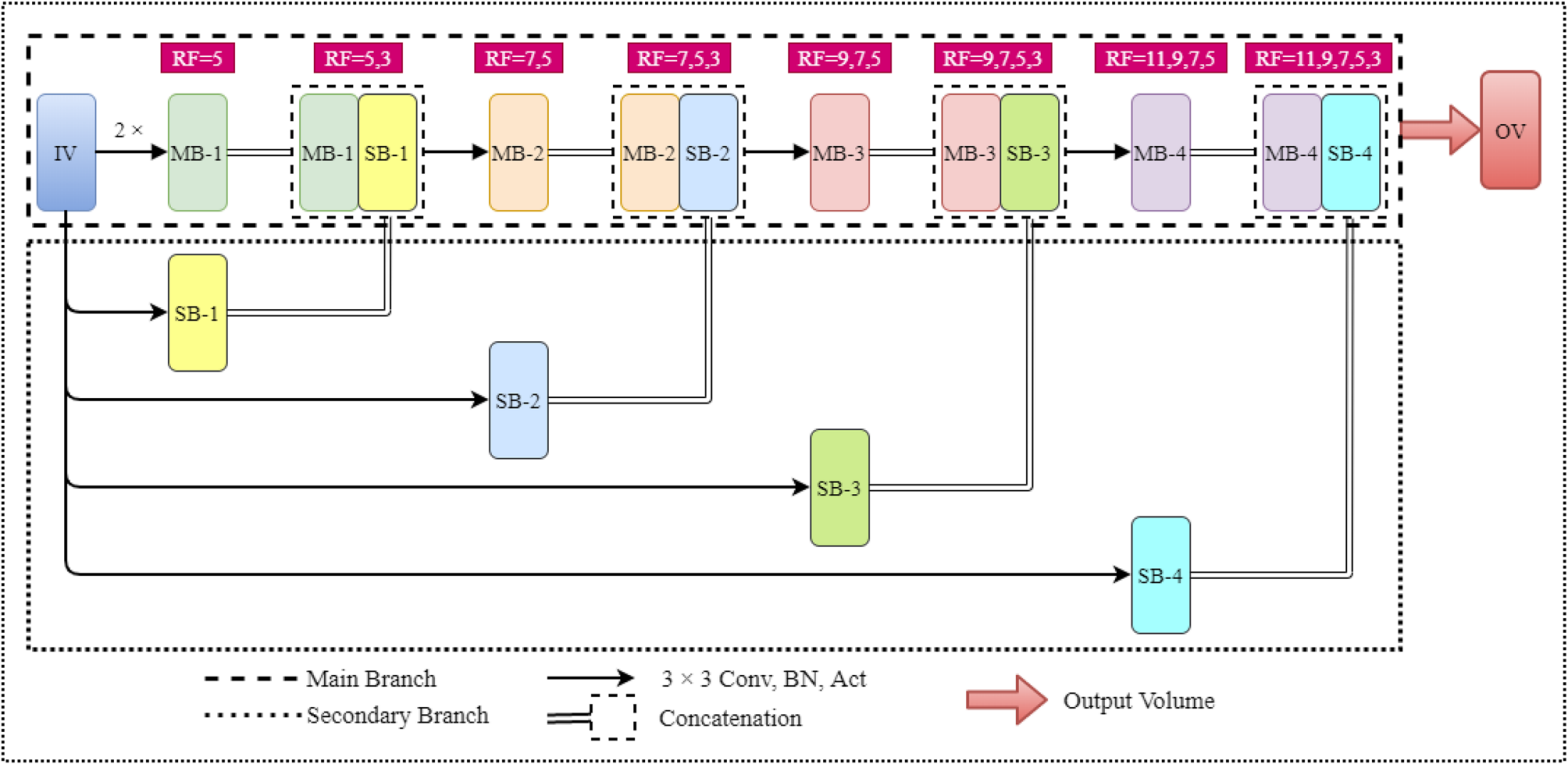
Thorough visual explanation of the proposed CMSFL-Net. IV and OV represent input and output volume, while RF, MB, and SB correspond to receptive field, main and secondary branch. Conv, BN, and Act stand for convolution operation, batch normalization, and activation function, respectively. All convolution operations are performed using 32 $3 \times 3$ filters.

Considering the information loss problem, we aim to learn as much useful information as possible from the input image before applying the max-pooling operation to address the aforementioned issue. For this purpose, we formulate a CMSFL module that benefits from several convolutional layers and concatenation operations. Figure 4 illustrates a detailed graphical overview of the CMSFL module.

The CMSFL module aims to extract as many useful features as possible from the input volume by applying a few convolutional layers with various receptive fields. Every convolution operation has a kernel size of 3$\times$3 and is followed by batch normalization (BN) and activation function. For smooth training and better generalizability, we employ weight initialization-based rectified linear unit activation function^50^. The other specification of the CMSFL module is that it considers the original features of the input volume to the module and concatenates the useful information from the secondary branch (SB) to every output (except the first convolution layer) of the main branch (MB). This concatenation operation helps retain a better representation of the useful features because the input volume to the CMSFL module exhibits the full information and features that are steadily lost when the convolution operations are applied. Therefore, to address the information loss, we consecutively concatenate output volumes of the convolution operation from the SB with the output feature maps from the MB.

3 $$\begin{aligned} O_V^{[l]} = Conv_{MB}(I_V)^\frown Conv_{SB}(I_V) \end{aligned}$$

**Figure 5:**
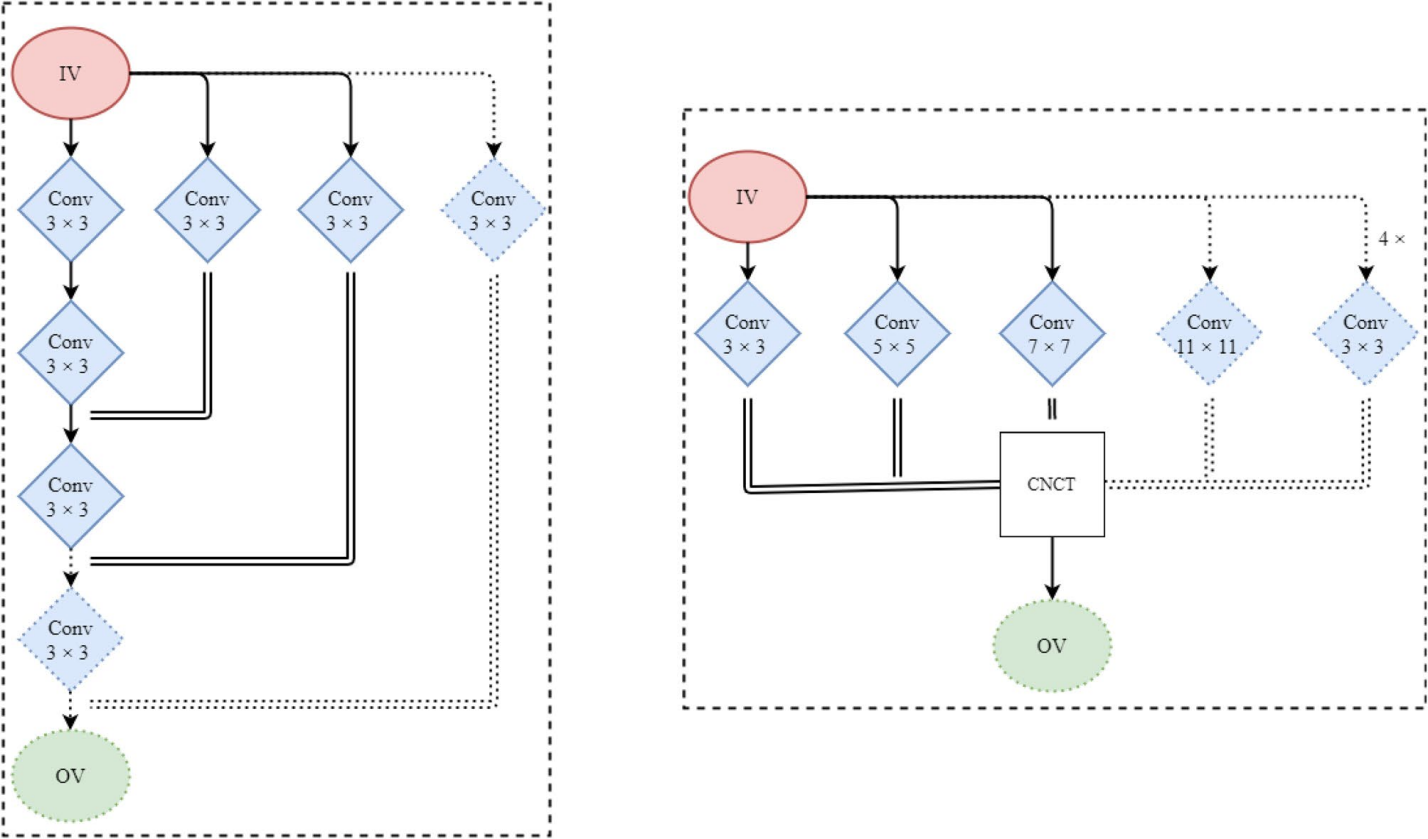
Comparison of the increasing RF size in convolutional layers in the proposed CMSFL module (left) and traditional methods (right). This is a sample to represent the number of convolution operations to increase the RF from (7, 5, 3) to (9, 7, 5, 3).

This also helps to efficiently increase the RF size of the convolution kernels. As can be seen, the RF size of the kernels in the convolution layers of the MB gradually increases as the training continues. The CMSFL module represents such an efficient approach to increasing RF size by applying only a single 3 $\times$ 3 convolution operation in SB and concatenating it with the output feature map from a 3 $\times$ 3 convolution operation in MB. A graphical illustration of the efficient way of RF size increase in the CMSFL module when compared to the traditional approaches can be seen in Fig. 5.

#### Increasing the RF

As shown in Fig. 5, to increase the RF from (7, 5, 3) to (9, 7, 5, 3), the traditional methods require four 3 $\times$ 3 convolution operations or a single 11 $\times$ 11 convolution operation. However, the same increase in the RF size can be achieved by employing only two 3 $\times$ 3 convolution operations in the proposed CMSFL module. The computation of total number of 3 $\times$ 3 convolution operations in the traditional and proposed methods are provided in Eqs. (4) and (5) as follows.

4 $$\begin{aligned}{} & {} \begin{aligned} RF:(3) \Longrightarrow& 3 \times 3 \rightarrow 1 \times [3 \times 3] \\ RF:(5, 3) \Longrightarrow& 5 \times 5 \rightarrow 2 \times [3 \times 3] \\ RF:(7, 5, 3) \Longrightarrow&7 \times 7 \rightarrow 3 \times [3 \times 3] \\ RF:(9, 7, 5, 3) \Longrightarrow& 11 \times 11 \rightarrow 4 \times [3 \times 3] \\ \end{aligned} \end{aligned}$$

5 $$\begin{aligned}{} & {} \begin{aligned} RF:(3) \Longrightarrow& 1 \times [3 \times 3] \\ RF:(5, 3) \Longrightarrow& 2 \times [3 \times 3] \\ RF:(7, 5, 3) \Longrightarrow& 2 \times [3 \times 3] \\ RF:(9, 7, 5, 3) \Longrightarrow& 2 \times [3 \times 3] \\ \end{aligned} \end{aligned}$$

From the Eqs. (4) and (5), the traditional methods require numerous convolution operations with a kernel size of $3 \times 3$ (or larger kernel sizes) to increase the RF in every other convolutional layer, while the proposed CMSFL module demands only two $3 \times 3$ convolution operations to increase the RF. Specifically, to obtain the RF of (9, 7, 5, 3), traditional methods require ten $3 \times 3$ convolutions while the proposed method can achieve the same RF size with only seven $3 \times 3$ convolution operations.

Moreover, the proposed CMSFL module requires significantly fewer trainable parameters and FLOPs that helps train the model efficiently, address overfitting, and achieve better generalizability to test data. The number of trainable parameters and FLOPs in an $l^{th}$ convolutional layer can be computed using the following equation.

6 $$\begin{aligned} \begin{aligned} P_{tr}^{[l]} =& ks^{[l]} \times cs^{[l-1]} \times cs^{[l]} + b^{[l]}\\ FLOPs^{[l]} = IV_{H}&\times IV_{W} \times cs^{[l-1]} \times ks^{[l]}\times ks^{[l]} \times cs^{[l]}\\ \end{aligned} \end{aligned}$$

In Eq. (6), *ks*, *cs*, and *b* correspond to kernel size, channel size, and bias, while $IV_{H}$ and $IV_{W}$ stand for input volume height and width, respectively. Thus, the traditional convolution operations need [$3 \times 3 + 5 \times 5 + 7 \times 7 + 11 \times 11$] weights multiplied by the number of filters in a specific layer *l* to achieve the RF of (9, 7, 5, 3). In contrast, the proposed model requires only [$7 \times$ $3 \times 3$] multiplied by the number of filters in layer *l* to obtain the aforementioned RF.

#### Loss function

Considering that the proposed method can be employed while training using datasets exhibiting a data imbalance problem, we implement a loss function that alleviates the aforementioned issue. Specifically, the UCIR loss function sets larger and smaller weights for over-represented and under-represented classes, respectively by $l^2$ normalizing both the weights and the activation. This means employing the cosine similarity rather than the dot product. For each class *c*, the last layer is changed as follows:

7 $$\begin{aligned} \omega _c = \frac{\exp (\alpha \cos (\varepsilon _c)}{\sum _j{\exp (\alpha \cos (\varepsilon _c)}} \\ \end{aligned}$$

In Eq. (7), $\alpha$, $\varepsilon$, and $\cos ()$ are learned scaling parameter, the last layer weights for the class *c*, and cosine similarity, respectively. After addressing the class imbalance problem, we formulate the loss function for training the proposed method. For this purpose, we employ weighted categorical cross-entropy loss. This loss function is formulated as follows:

8 $$\begin{aligned} \begin{aligned} L_{f} = -\frac{1}{M} \sum _{j=1}^{J}\sum _{i=1}^{M}\omega _j \times y_i^j \times \log (DCNN(x_i, j)) \end{aligned} \end{aligned}$$

In (8), *M*, *J*, $y_i^j$, $x_i$, and *DCNN* are the total number of images, classes, and ground truth for a training example *i* for class *j*, $i^th$ training image, and deep convolutional neural network, respectively.

In general, the data learning process aims to extract as many useful features as possible until the information is lost from the original image during convolution and max-pooling operations.

### Inference

After completing the second step of the proposed system and obtaining a trained CMSFL-Net model, we can classify the images using the this model in an inference stage. In this stage, the raw data should pass through the same pre-processing operations, as in the training stage, except for data augmentation. Specifically, a test set of a dataset or real-life images are represented as tensors, precisely resized, and standardized using (1). For standardization, *X* must be the training data, i.e., the same data that was used in the training and validation stages, to ensure that data in the inference stage follow the same distribution. The images are then input into the trained model, which consequently classifies them into one of the pre-defined categories.

## Experiments and results

In this section, we illustrate details of the conducted experiments and share the results by comparing the performance of the proposed system with existing state-of-the-art methods.

### Benchmarking datasets

To illustrate the excellent generalizability of the proposed method, we tested the performance of the CMSFL-Net using various real-life datasets that contain small-scale and large-scale images, datasets with a limited number of images. The overall information on the datasets can be seen in Table 1.

**Table 1:** General information on the datasets for the experiments.

Dataset	Dataset	Image	Image	Number of images		
Name	Type	Category	Size	Train	Validation	Test
CIFAR-10^51^	Small-scale	Objects	32 $\times$ 32	50,000	8000	2000
STL-10^52^	Small-scale	Objects	96 $\times$ 96	5000	6,00	1500
ImageNet-100^53^	Large-scale	Objects	Various	93,782	4874	7500
COVID-CT^54^	Limited number	CT scans	Various	500	150	96
BreakHis*^55^	Limited number	Microscopy	700 $\times$ 460	1680	600	200
Br35H^56^	Limited number	MRI	Various	2100	600	300

*Benign images only.

### Training details

In this subsection, we provide detailed information about the conducted experiments, such as experimental setup, baseline methods, and evaluation metrics.

#### Experimental setup

We formulated the baseline and proposed methods using Python version 3.6.9 and PyTorch library version 1.4.0. We initialized the weight parameters using Gaussian distribution and did not use bias parameters. We used $L_{f}$ (discussed in “Loss function” section) as the minimizing function and Adam optimizer with $\eta =0.0001$ and $\gamma =0.9$ as the parameter optimizer for the proposed method. The experiments were conducted using 32 GB NVIDIA Tesla V100-SXM2 GPU with CUDA 10.0 and a mini-batch size of 64 and 16 for small-scale and large-scale datasets, respectively. The models were trained for 50 epochs because the considered methods converged within this period of training and did not show improvements in performance when training a greater number of epochs.

#### Evaluation metrics

We employ various evaluation metrics to assess the performance of the proposed model compared with one of the baseline methods from different angles. Specifically, we define accuracy score (*AS*) and F1 score (*F1*) for the evaluation of the model’s performance.

9 $$\begin{aligned} \begin{aligned} AS&= \frac{\sum _{i}^{M}{\hat{y}}_i == y_i}{\sum _{i}^{M}y_i}\\ F1&= \frac{2 \times TP / (TP + FP) \times TP / (TP + FN)}{TP / (TP + FP) + TP / (TP + FN)} \end{aligned} \end{aligned}$$

In Eq. (9), ${\hat{y}}_i$, $y_i$ denote predicted output and ground truth for $i^th$ image, while *TP*, *FP*, *FN* correspond to true positive, false positive, and false negative, respectively.

**Figure 6:**
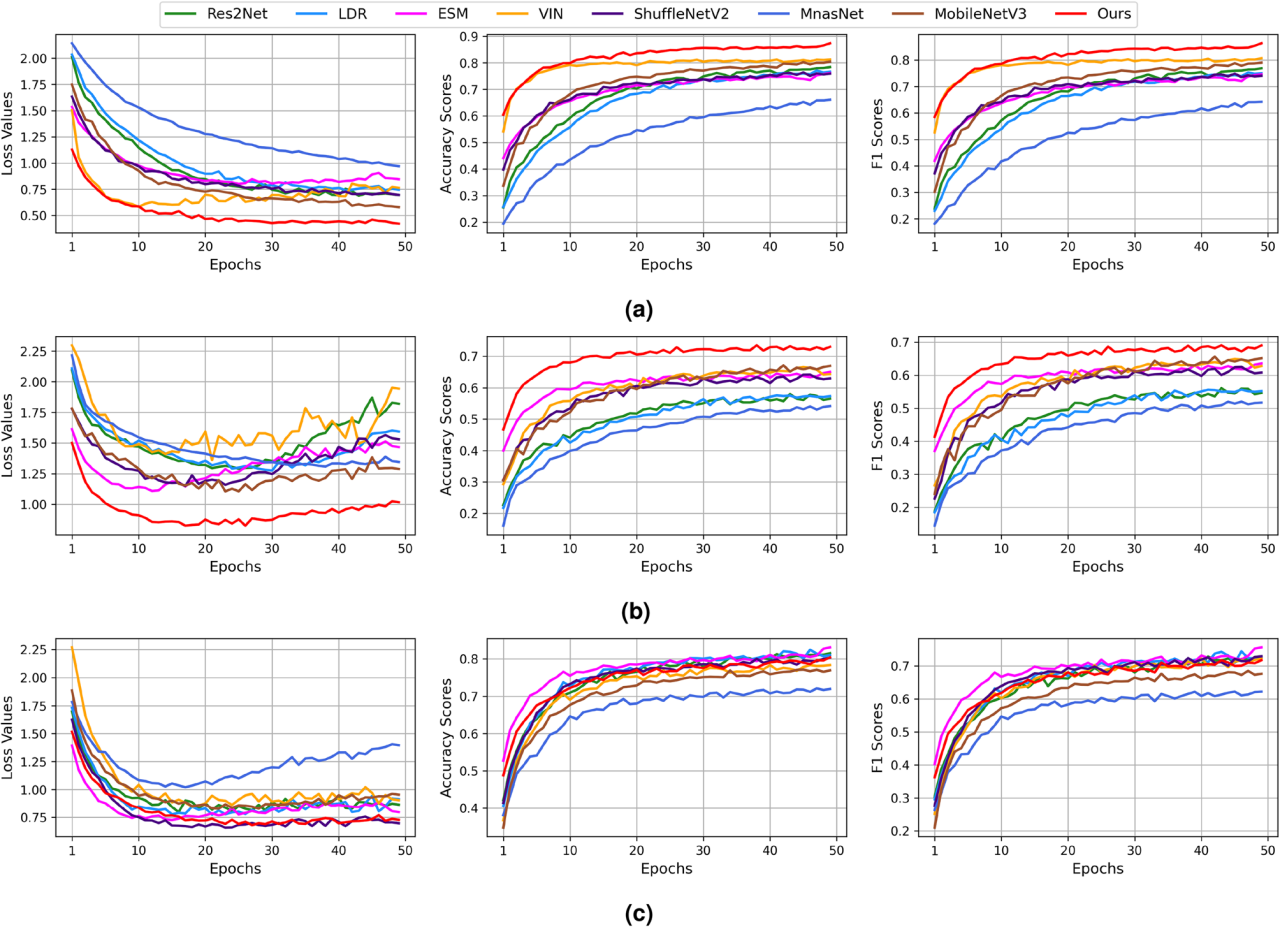
Experimental results using the baseline and proposed models on the validation set of the small-scale and large-scale datasets: (**a**) CIFAR-10, (**b**) STL-10, and (**c**) ImageNet100.

#### Baseline models

To show the efficiency of the proposed system, we selected two sorts of state-of-the-art methods, namely powerful methods that achieve high accuracy scores and lightweight methods that are efficient and fast. We compare the performance of the CMSFL-Net with the aforementioned models to show its good performance in terms of both efficiency and accuracy. As baseline models, we select Res2Net: A new multiscale backbone architecture (Res2Net)^34^, Learning Deformable Registration of Medical Images with Anatomical Constraints (LDR)^17^, Robust Image Classification Against Adversarial Attacks using Elastic Similarity Measures between Edge Count Sequences (ESM)^2^, Visual Interaction Networks (VIN)^12^, ShuffleNet V2^37^, MnasNet^38^, and MobileNetV3^39^. Because we discussed these methods in Section 2, we do not dive into the details of the aforementioned approaches in this section. All baseline models and the proposed method were trained and evaluated under the same circumstances as described in the next subsection.

### Experimental results with regards to computational efficiency

After formulating the baseline and proposed models for the experiments in accordance with the previous subsection, we compare them in terms of computation efficiency by focusing on the number of trainable parameters, FLOPs, model size, training, and inference time. The results of the comparison are shown in Table 2.

**Table 2:** Comparison of the baseline and proposed models in terms of computational efficiency, memory, and time*.

Model	Params	FLOPs	Size	Training	Inference
Name	(million)	(billion)	(mb)	(mins)**	(secs)
Res2Net	23.53	1.75	90.21	2.28	56.80
LDR	21.72	1.40	92.22	1.98	42.21
ESM	23.00	1.67	77.34	2.19	59.43
VIN	27.16	2.1	95.53	2.35	70.10
ShuffleNetV2	1.23	1.24	5.10	1.04	34.53
MnasNet	3.12	0.93	12.41	1.31	26.59
MobileNetV3	2.24	0.73	8.96	0.53	8.75
Ours	**0.86**	**0.65**	**3.44**	**0.47**	**7.53**

Number of trainable parameters, training, and inference time may differ depending on the datasets characteristics. *This information is based on experiments using 32 GB NVIDIA Tesla V100-SXM2 GPU. **Average training time per epoch.

Significant values are in [bold].

As indicated in Table 2, the proposed model significantly outperformed the powerful baseline models, such as Res2Net, LDR, ESM, and VIN, and achieved comparable performance when compared with the lightweight models, like ShuffleNetV2, MnasNet, and MobileNetV3. Specifically, the CMSFL-Net required considerably fewer trainable parameters by outperforming its closest peer, ShuffleNetV2, by approximately 30%. Moreover, the proposed model achieved the best performance in FLOPs and trained model size, too. To be more precise, CMSFL-Net exhibited up to 2 times fewer FLOPs in comparison with the powerful models, while its model size was only 3.44 MB, which is the smallest storage among the considered models. Regarding training and inference time, the proposed model was the fastest one when compared with the baseline models by demanding 0.47 minutes per epoch training and 7.53 seconds on training and testing sets of the BreakHis dataset, respectively.

### Experimental results on small-scale and large-scale image datasets

The results of the validation set of the CIFAR-10, STL-10, and ImageNet-100 datasets using accuracy-related metrics are provided in Fig. 6. For example, LDR showed a steady increase in AS and F1 as the training progressed; however, the model achieved the lowest accuracy-related scores in comparison to the other considered models on the aforementioned datasets. The baseline models achieved similar scores in loss, AS, and F1 on the CIFAR-10 and STL-10 datasets, where VIN and MobileNetV3 attained the second-best scores. The highest scores in AS and F1 in the small-scale image datasets were obtained by the proposed system. The CMSFL-Net significantly outperformed its peers by obtaining approximately 7% higher results when evaluated using AS and F1. On the large-scale ImageNet-100 dataset, the proposed method demonstrated stable performance during the training process and achieved the second-best results when assessed using accuracy-related metrics. Throughout the training, except for the several final epochs, the performance of the proposed method was similar to the best-performing ESM method, which exhibited a sudden increase in AS and F1 in the last epochs of training and outperformed the proposed system.

### Experimental results on datasets with limited number of data

**Figure 7:**
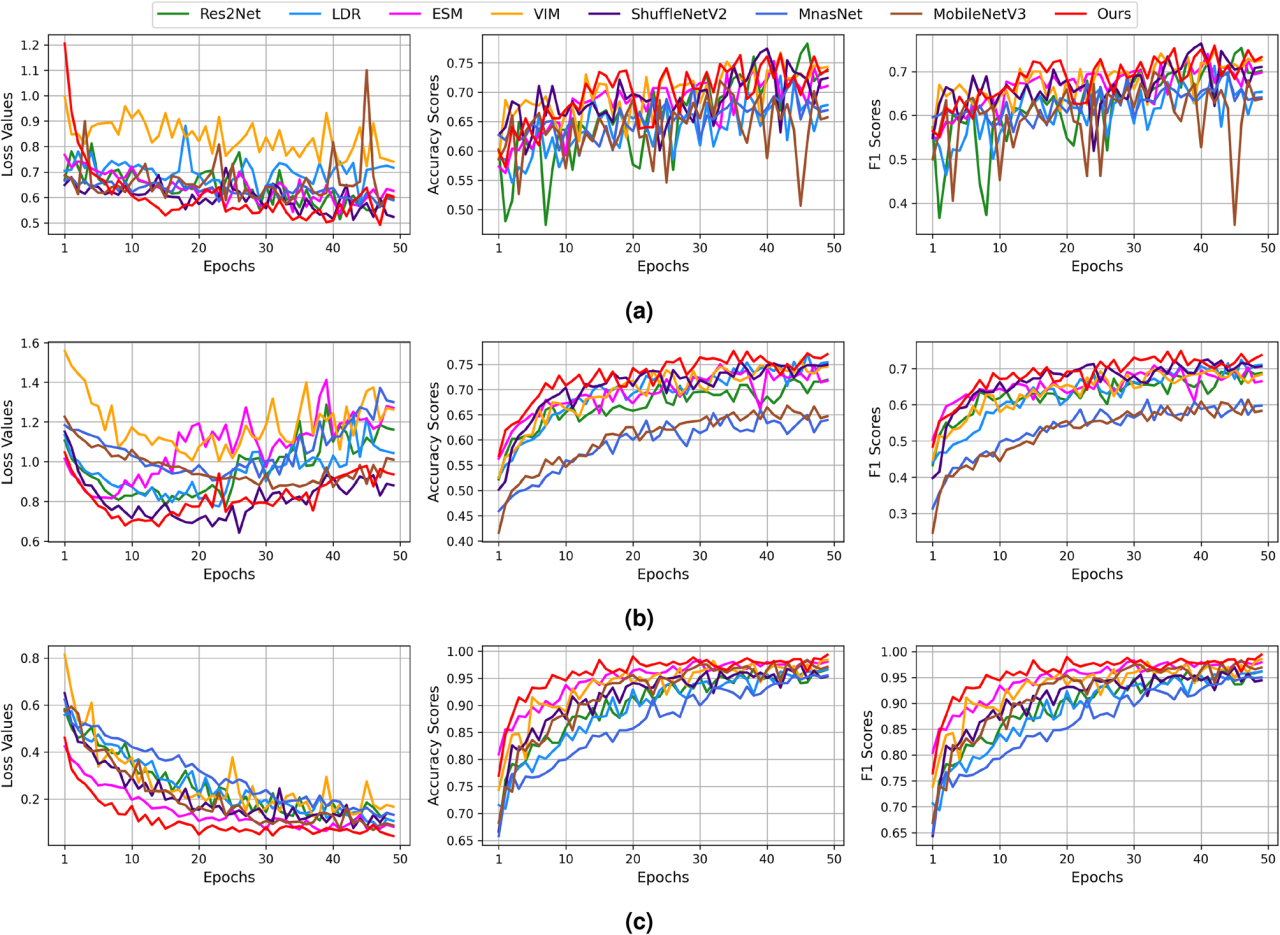
Experimental results using the baseline and proposed models on the validation set of the datasets with a limited number of data: (**a**) COVID-CT, (**b**) BreakHis, and (**c**) Br35H.

Figure 7 shows the comparison of the model’s performance on the validation set of the COVID-CT, BreakHis, and Br35H datasets when assessed with loss value, AS, and F1.

In fact, the results of the accuracy-related metrics are very noisy because the datasets exhibited a small number of training data that results in fluctuations in training. Overall, the performance of the baseline models such as VIM, ShuffleNetV2, Res2Net, and ESM was comparable with one of the proposed models, while MnasNet and LDR methods obtained significantly lower AS and F1 when compared with the CMSFL-Net. Overall, the proposed method obtained the optimal results in AS by reaching 0.732, 0.995, and 0.773 on the validation set of the COVID-CT, BreakHis, and Br35H datasets, respectively. Regarding the F1, the results of the proposed method were similar to the ones when assessed using AS evaluation metric, and it achieved the F1 of 0.733, 0.993, and 0.736, respectively.

## Discussion

This section discusses the results of the conducted experiments using the test sets of the considered datasets and shares the results of ablation studies. Moreover, it exhibits a qualitative comparison of the baseline and proposed methods and enumerates the limitations of the proposed method.

### Generalizability of the considered models on the small and large-scale image datasets

We tested the performance of the baseline and proposed methods on the test set of the considered datasets to compare their generalization ability on unseen data during inference in terms of loss, AS, and F1. The results of the experiments are shown in Table 3.

**Table 3:** Comparison of the baseline and proposed models on the test sets of the small- and large-scale image datasets in terms of loss and accuracy*.

Datasets	Models	Loss	AS	F1
CIFAR-10	Res2Net	1.155 ± 0.073	0.788 ± 0.009	0.760 ± 0.011
	LDR	1.694 ± 0.085	0.688 ± 0.013	0.659 ± 0.010
	ESM	**1.040 ± 0.017**	0.775 ± 0.010	0.756 ± 0.007
	VIN	2.070 ± 0.123	0.760 ± 0.012	0.715 ± 0.017
	ShuffleNetV2	1.220 ± 0.029	0.755 ± 0.015	0.706 ± 0.018
	MnasNet	2.588 ± 0.141	0.611 ± 0.034	0.597 ± 0.040
	MobileNetV3	1.479 ± 0.053	0.731 ± 0.012	0.692 ± 0.016
	Ours	1.146 ± 0.066	**0.794 ± 0.023**	**0.769 ± 0.019**
STL-10	Res2Net	1.948 ± 0.069	0.620 ± 0.011	0.600 ± 0.016
	LDR	2.001 ± 0.073	0.612 ± 0.019	0.587 ± 0.022
	ESM	1.678 ± 0.013	0.658 ± 0.014	0.644 ± 0.010
	VIN	1.530 ± 0.093	0.735 ± 0.010	0.716 ± 0.008
	ShuffleNetV2	1.895 ± 0.073	0.658 ± 0.007	0.643 ± 0.011
	MnasNet	2.163 ± 0.199	0.520 ± 0.018	0.504 ± 0.014
	MobileNetV3	1.723 ± 0.036	0.669 ± 0.010	0.661 ± 0.08
	Ours	**1.137 ± 0.024**	**0.735 ± 0.007**	**0.723 ± 0.010**
ImageNet-100	Res2Net	0.863 ± 0.083	0.835 ± 0.009	0.757 ± 0.006
	LDR	0.896 ± 0.090	0.852 ± 0.011	0.775 ± 0.013
	ESM	**0.837 ± 0.102**	**0.874 ± 0.016**	**0.809 ± 0.018**
	VIN	0.894 ± 0.067	0.841 ± 0.007	0.781 ± 0.006
	ShuffleNetV2	0.902 ± 0.049	0.847 ± 0.010	0.777 ± 0.008
	MnasNet	1.431 ± 0.093	0.744 ± 0.014	0.652 ± 0.017
	MobileNetV3	1.312 ± 0.048	0.762 ± 0.005	0.654 ± 0.007
	Ours	0.852 ± 0.013	0.863 ± 0.012	0.801 ± 0.013

Lower loss and higher AS and F1 scores correspond to better performance of a model. *This information is based on experiments using 32 GB NVIDIA Tesla V100-SXM2 GPU

Significant values are in [bold].

As presented in the table, the proposed system outperformed the baseline methods in both small-scale datasets, CIFAR-10 and STL-10, except for the loss value on the CIFAR-10, where the ESM method achieved the lowest score by obtaining 1.040, which is only 0.6% better than the one of the proposed method. In other accuracy-related metrics, the proposed model obtained better generalization to the unseen data. On the large-scale ImageNet-100 dataset, the proposed method achieved the second-best performing results by obtaining 1,79%, 1,25%, and 0,99% lower scores than the best performing ESM method in loss, AS, and F1, respectively. Considering that the ESM model is a computationally expensive network, the proposed method demonstrated the best accuracy-efficiency trade-off in the most commonly used ImageNet benchmarking dataset.

### Generalizability of the considered models on the datasets with a limited number of data

Table 4 shows the experimental results of the inference step on the test sets of the COVID-CT, BreakHis, and BrH35 datasets.

**Table 4:** Comparison of the baseline and proposed models on the test sets of the datasets with limited training data in terms of loss and accuracy*.

Datasets	Models	Loss	AS	F1
COVID-CT	Res2Net	0.521 ± 0.020	0.812 ± 0.003	0.777 ± 0.005
	LDR	0.447 ± 0.016	0.740 ± 0.008	0.717 ± 0.006
	ESM	**0.367 ± 0.020**	**0**.**896** ± 0.007	**0.863 ± 0.010**
	VIN	0.673 ± 0.013	0.812 ± 0.003	0.793 ± 0.005
	ShuffleNetV2	0.791 ± 0.018	0.688 ± 0.010	0.662 ± 0.013
	MnasNet	0.471 ± 0.095	0.771 ± 0.006	0.760 ± 0.010
	MobileNetV3	0.568 ± 0.008	0.740 ± 0.005	0.727 ± 0.008
	Ours	0.478 ± 0.012	0.828 ± 0.004	0.803 ± 0.002
BreakHis	Res2Net	1.315 ± 0.018	0.758 ± 0.009	0.713 ± 0.007
	LDR	1.694 ± 0.020	0.688 ± 0.004	0.659 ± 0.008
	ESM	1.378 ± 0.031	0.764 ± 0.010	0.698 ± 0.008
	VIN	2.070 ± 0.089	0.760 ± 0.027	0.715 ± 0.023
	ShuffleNetV2	1.221 ± 0.023	0.755 ± 0.011	0.706 ± 0.007
	MnasNet	2.588 ± 0.359	0.611 ± 0.059	0.597 ± 0.070
	MobileNetV3	1.478 ± 0.013	0.731 ± 0.007	0.692 ± 0.010
	Ours	**1.166 ± 0.010**	**0.769 ± 0.003**	**0.729 ± 0.005**
Br35H	Res2Net	0.056 ± 0.010	0.980 ± 0.002	0.969 ± 0.004
	LDR	0.176 ± 0.015	0.933 ± 0.006	0.932 ± 0.005
	ESM	0.048 ± 0.007	0.987 ± 0.006	0.984 ± 0.002
	VIN	0.087 ± 0.013	0.977 ± 0.009	0.973 ± 0.006
	ShuffleNetV2	0.078 ± 0.011	0.980 ± 0.005	0.975 ± 0.003
	MnasNet	0.073 ± 0.017	0.978 ± 0.004	0.973 ± 0.004
	MobileNetV3	0.674 ± 0.009	0.980 ± 0.010	0.974 ± 0.008
	Ours	**0.039 ± 0.008**	**0.991 ± 0.002**	**0.990 ± 0.004**

Lower loss and higher AS and F1 scores correspond to better performance of a model. *This information is based on experiments using 32 GB NVIDIA Tesla V100-SXM2 GPU.

Significant values are in [bold].

The table shows that ESM achieves the lowest loss and the highest accuracy on the COVID-CT dataset by significantly outperforming its peers in terms of generalizability to the unseen data. The proposed method attains satisfactory results by ranking second on the aforementioned dataset. However, on the other two medical image datasets, namely BreakHis and Br35H, the proposed method obtains the best scores in terms of the evaluation metrics: loss, AS, and F1. Specifically, the proposed method achieves perfect accuracy on the test set of Br35H by reaching 0.991 and 0.990 in AS and F1, respectively. In general, the proposed approach shows good efficiency and better generalizability to unseen data than its peers.

### Ablation studies of the CMSFL-Net

We also conducted extensive ablation studies and tested different versions of the proposed method to determine the best trade-off between speed and accuracy. The results of the studies are shown in Table 5.

**Table 5:** Ablation studies of the proposed model on the test sets of the considered datasets*.

Datasets	Models	Loss	AS	F1	TP	TT	IT
CIFAR-10	CMSFL-Net (4)	1.56 ± 0.06	0.73 ± 0.04	0.71 ± 0.03	0.56	87.33	6.87
	CMSFL-Net (6)	*1.15 ± 0.01*	*0.79 ± 0.02*	*0.77 ± 0.02*	*0.86*	*93.21*	*32.35*
	CMSFL-Net (8)	1.13 ± 0.02	0.80 ± 0.01	0.78 ± 0.01	1.17	98.11	35.01
	CMSFL-Net (10)	1.20 ± 0.01	0.78 ± 0.02	0.75 ± 0.01	1.49	105.10	37.12
STL-10	CMSFL-Net (4)	1.43 ± 0.01	0.69 ± 0.01	0.66 ± 0.01	0.56	11.23	9.81
	CMSFL-Net (6)	*1.14 ± 0.02*	*0.74 ± 0.01*	*0.72 ± 0.10*	*0.86*	*14.11*	*10.29*
	CMSFL-Net (8)	1.10 ± 0.01	0.75 ± 0.01	0.72 ± 0.01	1.17	16.99	10.63
	CMSFL-Net (10)	1.21 ± 0.01	0.72 ± 0.01	0.70 ± 0.01	1.49	18.21	11.32
ImageNet-100	CMSFL-Net (4)	1.12 ± 0.02	0.79 ± 0.01	0.74 ± 0.01	0.56	1,987.16	109.18
	CMSFL-Net (6)	*0.85 ± 0.01*	*0.86 ± 0.01*	*0.80 ± 0.01*	*0.86*	*2,366.42*	*134.49*
	CMSFL-Net (8)	0.83 ± 0.02	0.86 ± 0.02	0.81 ± 0.01	1.17	2,719.61	148.18
	CMSFL-Net (10)	0.91 ± 0.01	0.83 ± 0.01	0.79 ± 0.01	1.49	3,101.83	162.19
COVID-CT	CMSFL-Net (4)	0.87 ± 0.02	0.76 ± 0.01	0.72 ± 0.01	0.56	69.09	11.20
	CMSFL-Net (6)	*0.48 ± 0.01*	*0.83 ± 0.01*	*0.80 ± 0.01*	*0.86*	*80.92*	*13.56*
	CMSFL-Net (8)	0.53 ± 0.01	0.84 ± 0.01	0.81 ± 0.01	1.17	91.96	17.18
	CMSFL-Net (10)	0.59 ± 0.02	0.83 ± 0.02	0.80 ± 0.02	1.49	100.81	23.05
BreakHis	CMSFL-Net (4)	1.51 ± 0.01	0.70 ± 0.02	0.68 ± 0.02	0.56	198.91	7.37
	CMSFL-Net (6)	*1.17 ± 0.01*	*0.77 ± 0.01*	*0.73 ± 0.10*	*0.86*	*235.22*	*9.45*
	CMSFL-Net (8)	1.21 ± 0.01	0.76 ± 0.01	0.73 ± 0.01	1.17	299.12	15.12
	CMSFL-Net (10)	1.25 ± 0.01	0.74 ± 0.01	0.71 ± 0.01	1.49	318.41	19.11
Br35H	CMSFL-Net (4)	0.08 ± 0.01	0.97 ± 0.01	0.94 ± 0.01	0.56	67.91	4.63
	CMSFL-Net (6)	*0.04 ± 0.01*	*0.99 ± 0.01*	*0.99 ± 0.01*	*0.86*	*78.95*	*5.57*
	CMSFL-Net (8)	0.05 ± 0.01	0.99 ± 0.01	0.99 ± 0.01	1.17	91.72	7.10
	CMSFL-Net (10)	0.07 ± 0.01	0.98 ± 0.01	0.97 ± 0.01	1.49	102.98	9.61

Lower loss and higher AS and F1 scores correspond to better performance of a model. TP, TT, and IT correspond to trainable parameters (millions), average training time per epoch (seconds), and inference time (seconds), respectively. *This information is based on experiments using 32 GB NVIDIA Tesla V100-SXM2 GPU.

Significant values are in [italics].

In the table, we modified the network using a different number of CMSFL modules ranging from 4 to 10 and compared the results using loss, AS, and F1 evaluation metric scores and the number of trainable parameters, training time, and inference time. Overall, it is shown that a network with the fewest number of CMSFL modules was faster in training and inference but achieved lower accuracy on the test sets of the considered datasets. When the number of CMSFL modules was increased by two, CMSFL-Net (refer to Section ) showed a significant decrease in loss and an increase in accuracy-related metrics. However, increasing the number of CMSFL modules did not provide significant improvements in the performance of the proposed method. Although there was a slight increase in accuracy with the proposed model with 8 CMSFL modules, it resulted in a considerable increase in training and inference time. Increasing the CMSFL modules to ten decreases the network performance. Considering these findings, we selected the proposed model architecture with six CMSFL modules as a default network because it achieved the best accuracy and speed trade-off in the conducted ablation studies.

## Data Availability

All data generated or analyzed in this study are included in this published article. All six datasets used are publicly available datasets. All of them are cited in the paper according to the rules of conducting research. The citations and the datasets can be found in the “Benchmarking datasets” subsection of the “Experiments and Results” section of the paper.
